# Cross‐Cohort Transcriptomic and Oral Microbiome Analyses Identify Ferroptosis‐Associated Signatures in Periodontitis and Oral Squamous Cell Carcinoma

**DOI:** 10.1155/ijog/7538825

**Published:** 2026-11-03

**Authors:** Chenguang Qiu, Peng She, Lin Wu

**Affiliations:** ^1^ Department of Stomatology Affiliated People′s Hospital of Jiangsu University Zhenjiang City Jiangsu Province China

**Keywords:** cell composition, cross-cohort analysis, ferroptosis-associated gene signature, GEO database, oral microbiome, oral squamous cell carcinoma, periodontitis, SAT1, single-cell RNA sequencing

## Abstract

**Background:**

Periodontitis (PD) and oral squamous cell carcinoma (OSCC) have been investigated in relation to inflammation, oral microbial alterations, and iron/redox biology. Whether ferroptosis‐associated expression patterns overlap across independently sampled periodontal and cancer cohorts remains uncertain. We characterized these patterns without presuming that the available datasets represent matched individuals or a temporal disease sequence.

**Methods:**

We analyzed gingival single‐cell RNA‐sequencing data (GSE171213), an OSCC discovery expression cohort (GSE30784), and an independent oral tongue squamous cell carcinoma validation cohort (GSE13601). Periodontal microbial functional gene profiles (GSE54290) and a cross‐study OSCC 16S abundance resource were examined separately. Analyses included cell annotation, KEGG ferroptosis gene‐set scoring, sample‐ and cell type‐level comparisons, differential expression, external transcriptomic signature validation, microbial diversity and abundance analyses, and LPS‐stimulated cell‐line RT‐qPCR and ELISA measurements. The transcriptomic and microbial cohorts were not linked at the individual‐patient level.

**Results:**

Quality control retained 34,342 cells from 12 gingival samples (four healthy controls [HCs], five PD, and three treated periodontitis [PDT]), assigned to 11 annotated cell populations. Mean sample‐level ferroptosis gene‐set scores were 0.081 in HC, 0.114 in PD, and 0.076 in PDT; these descriptive scores do not measure ferroptotic cell death. SAT1 showed a positive PD‐versus‐HC association in cell type pseudobulk analysis (log2 fold change, 0.546; false discovery rate [FDR], 0.012) and positive effects across all three transcriptomic cohorts. A discovery‐derived ferroptosis‐associated OSCC signature achieved an area under the receiver operating characteristic curve of 0.933 in GSE13601. Periodontal functional profiling identified 25 advanced PD‐associated annotations (FDR < 0.05), including lower ferrochelatase signals. Cross‐study OSCC microbiome analysis found 293 diagnosis‐associated features after project adjustment; project‐held‐out classification yielded an AUC of 0.674. Following lipopolysaccharide stimulation, GPX4, SLC7A11, and FTH1 increased and ACSL4 decreased at the measured mRNA and protein levels in gingival fibroblasts and THP‐1‐derived macrophages.

**Conclusions:**

Independently sampled periodontal and OSCC cohorts show ferroptosis‐associated transcriptomic changes, with SAT1 emerging as a cross‐cohort candidate. Parallel microbial datasets identify disease‐associated functional gene and taxonomic differences but do not demonstrate within‐patient host–microbe interactions or causal mediation. LPS‐exposed cell‐line measurements show expression changes in selected ferroptosis‐associated markers, not ferroptotic cell death or functional resistance. The results are exploratory associations; in the absence of direct ferroptosis assays, paired longitudinal cohorts, and prospective clinical validation, they cannot establish a disease‐progression mechanism, clinically deployable biomarker or therapeutic efficacy.

## 1. Introduction

Periodontitis (PD) is a common inflammatory disease that can progressively destroy tooth‐supporting tissues and contribute to tooth loss. A systematic review estimated the global age‐standardized prevalence of severe PD at 11.2% in 2010 [1]. This multifactorial condition affects the gingiva, periodontal ligament, cementum, and alveolar bone and involves dysbiotic subgingival biofilms and dysregulated host immune responses [2, 3]. Beyond oral health, PD has been associated with cardiovascular disease, although the observational association does not establish causality [4].

The pathogenesis of PD involves complex interactions between periodontal pathogens and host immune cells. Periodontal‐associated species such as *Porphyromonas gingivalis*, *Tannerella forsythia*, and *Treponema denticola* contribute to polymicrobial dysbiosis and host inflammatory responses [5]. The subsequent recruitment and activation of immune cells, including macrophages, neutrophils, T cells, and B cells, leads to the release of proinflammatory cytokines and tissue‐degrading enzymes that perpetuate tissue destruction [6, 7]. Understanding the molecular mechanisms underlying this inflammatory cascade is essential for developing targeted therapeutic strategies.

Ferroptosis is a recently discovered form of regulated cell death characterized by iron‐dependent lipid peroxidation and distinct from apoptosis, necrosis, and autophagy [8]. The execution of ferroptosis is driven by the accumulation of lipid reactive oxygen species (ROS) when cellular antioxidant capacity is overwhelmed. Glutathione peroxidase 4 (GPX4) serves as the central negative regulator of ferroptosis by reducing lipid hydroperoxides to nontoxic lipid alcohols [9]. The cystine/glutamate antiporter system Xc‐, encoded by SLC7A11, provides cystine for glutathione synthesis, thereby supporting GPX4 function and preventing ferroptosis [10]. Additional regulators include ferritin heavy chain (FTH1), involved in iron storage, and ACSL4, involved in lipid composition and ferroptosis susceptibility [11].

Emerging research links ferroptosis‐associated pathways with inflammation and tissue injury [12, 13]. In PD, bacterial challenge, inflammatory responses, and changes in iron/redox handling provide a rationale for investigating these pathways, but pathway‐associated expression alone cannot establish ferroptotic death. A study of gingival crevicular fluid from patients monitored over 12 weeks identified proteins associated with both clinical progression and ferroptosis‐related biology, providing associative rather than direct evidence of ferroptotic cell death [14]. The contribution of ferroptosis to periodontal pathology and its relationship with individual immune cell populations therefore remain incompletely established.

The periodontal tissue environment includes immune cells, fibroblasts, epithelial cells, and endothelial cells, as characterized in the original single‐cell study underlying GSE171213 [15]. Changes in the proportions or transcriptional states of these populations may accompany inflammation, although cell composition estimates alone cannot establish immune‐cell recruitment, treatment response, or causal effects. Characterizing cell type‐associated expression patterns can help define questions for targeted validation.

Single‐cell RNA sequencing (scRNA‐seq) enables cell population annotation and assessment of expression heterogeneity in complex tissues [16]. Biological donors, rather than individual cells, remain the independent units for between‐patient inference [17]. A related interpretive distinction applies to ferroptosis gene‐set scores: These quantify relative expression of pathway‐associated genes, including genes with opposing functional roles, and cannot by themselves demonstrate iron‐dependent cell death.

PD and oral squamous cell carcinoma (OSCC) can be studied in relation to overlapping inflammatory and redox‐associated processes, but an association across separate datasets cannot demonstrate progression from one disease to the other. We therefore used two complementary, unpaired analytical tracks: gingival single‐cell and OSCC transcriptomes to investigate ferroptosis‐associated gene expression patterns and periodontal functional gene and OSCC 16S microbiome datasets to describe disease‐associated microbial features. Our primary reporting objective was to characterize the periodontal cell landscape and ferroptosis‐associated expression changes; independent OSCC signature validation and separate microbiome analyses provided additional evidence. Cell trajectories, ligand–receptor coexpression, and cross‐cohort candidate ranking were exploratory and hypothesis‐generating. These analyses do not constitute matched‐patient multiomics integration, direct host–microbiome correlation, causal mediation, or chronological reconstruction of a PD‐to‐OSCC transition.

## 2. Methods

### 2.1. Data Sources and Study Design

Public human datasets were assembled into two coordinated analytical tracks: a three‐cohort transcriptomic analysis and disease‐specific microbial analyses. Gingival scRNA‐seq data were obtained from GSE171213. GSE30784 served as the OSCC discovery transcriptome cohort, and GSE13601 served as an external oral tongue squamous cell carcinoma (SCC) validation cohort. Periodontal microbial functional gene profiles were obtained from GSE54290. A cross‐study oral 16S abundance table and metadata were retrieved from the OSCC meta‐analysis repository for the OSCC microbiome analysis. KEGG pathway hsa04216 defined the ferroptosis gene set, and Greengenes 13.8 supplied 99% operational taxonomic unit annotations. All hypothesis tests were two‐sided, and *p* values were adjusted by the Benjamini–Hochberg procedure where multiple features were tested. Fixed‐seed procedures used Seed 42.

### 2.2. Transcriptomic Analyses

The 12 GSE171213 count matrices represented four healthy control (HC), five PD, and three treated periodontitis (PDT) samples. Gene‐by‐cell tables were converted to sparse cell‐by‐gene matrices. Cells expressing 200–5000 genes, with at least 500 total counts and less than 20% mitochondrial transcripts, were retained; genes detected in fewer than three cells were removed. Counts were normalized to 10,000 per cell and log1p transformed. Three thousand highly variable genes were selected with sample as the batch key. Principal component analysis was followed by a 15‐nearest‐neighbor graph using 35 components, UMAP embedding (minimum distance, 0.35), and Leiden clustering (resolution, 0.7). Cluster markers were ranked with the Wilcoxon test, and cell identities were assigned from the overlap between each cluster′s Top 80 markers and canonical lymphoid, myeloid, stromal, and epithelial marker sets.

Ferroptosis activity was quantified from the KEGG hsa04216 genes detected in the single‐cell matrix using Scanpy′s matched control gene‐scoring procedure. For cell type‐resolved comparisons, gene expression and ferroptosis scores were averaged within each sample and cell type before PD‐versus‐HC Welch tests, followed by FDR correction. Diffusion pseudotime was estimated in the endothelial compartment; graph construction used a fixed‐seed subsample when the compartment exceeded 12,000 cells, and the HC cell with the lowest ferroptosis score was selected as the root. For the ligand–receptor pairs examined, sender–receiver coexpression was summarized as the product of mean ligand expression in the sender population and mean receptor expression in the receiver population. For Figure 1E, PD coexpression scores were log1p transformed for visualization; Figure 1F compares the PD‐minus‐HC difference in maximum raw coexpression across pairs.

**Figure 1:**
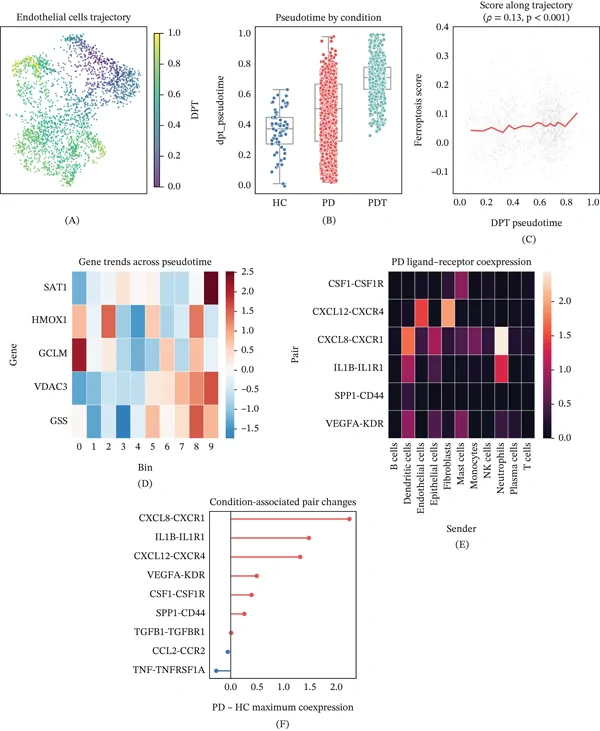
Endothelial trajectory and ligand–receptor coexpression. (A) Endothelial cell UMAP colored by diffusion pseudotime. (B) Distribution of endothelial pseudotime across HC, PD, and PDT. (C) Cell‐level ferroptosis scores plotted against pseudotime; the red line shows the mean score in 20 pseudotime bins. (D) *Z*‐scaled expression trajectories of the five leading ferroptosis‐associated genes across 10 pseudotime bins. (E) Log1p‐transformed maximum ligand–receptor coexpression scores in PD, shown for the six strongest pairs and their sender populations. (F) Difference in maximum coexpression between PD and HC for the 9 pairs with the largest absolute changes. DPT, diffusion pseudotime.

Embedded GEO expression matrices and platform annotations were parsed for GSE30784 (GPL570) and GSE13601 (GPL8300), the latter representing an oral tongue SCC validation cohort. GSE13601 intensities were transformed as log2(*x* + 1), whereas GSE30784 was retained on its deposited log‐compatible scale. Probes were mapped to gene symbols, retaining the probe with the largest across‐sample variance when several probes represented the same gene. Differential expression was assessed by Welch tests with FDR correction. A ferroptosis signature was derived in GSE30784 from the direction of KEGG ferroptosis genes significant at FDR < 0.05. Retained genes were standardized within each cohort, multiplied by their discovery‐cohort directions and averaged. The direction of the score was fixed in GSE30784 and applied unchanged to GSE13601. External discrimination was calculated from the unaltered validation scores by receiver operating characteristic analysis, and genome‐wide effect concordance was evaluated by the Spearman correlation.

### 2.3. Microbiome and Integrative Analyses

The oral periodontal subset of GSE54290 comprised healthy, moderate PD, and advanced PD samples. HuMiChip intensities were transformed as log2(*x* + 1), mapped to GPL18196 functional gene annotations, and averaged across repeated annotation names. Advanced PD was compared with HCs by Welch testing with FDR correction. Functional categories were assigned by keyword matching against the deposited platform annotations. The OSCC microbiome analysis used the ConQuR‐adjusted table rarefied to 17,794 reads. Greengenes taxonomy was joined by feature identifier, and OSCC and HC relative abundances were centered‐log‐ratio transformed using a 10−6 pseudocount. Differential abundance was estimated in linear models containing diagnosis and project indicators.

Microbial classification was evaluated by five‐fold stratified group cross‐validation, with complete projects held out from model training. Within each training fold, the 100 features with the lowest diagnosis‐associated *p* values among nonzero variance features were selected, and a 500‐tree class‐balanced random forest with a minimum leaf size of three was fitted. The overall area under the curve (AUC) was calculated from out‐of‐fold predictions. For the three‐cohort transcriptomic evidence ranking, ferroptosis gene effects from sample‐level PD single‐cell summaries, GSE30784 and GSE13601 were aligned by gene symbol. Each gene was ranked by the number of the three transcriptomic cohorts in which FDR was below 0.05 plus the proportion of available effects that shared the dominant direction.

### 2.4. Cell Culture and Lipopolysaccharide (LPS) Stimulation

Human gingival fibroblasts (HGFs) were obtained from the American Type Culture Collection (ATCC). Cells were cultured in Dulbecco′s Modified Eagle Medium (DMEM) supplemented with 10% fetal bovine serum (FBS) and 1% penicillin‐streptomycin at 37°C in a humidified atmosphere containing 5% CO_2_. THP‐1 human monocytic cells were also obtained from ATCC and maintained in RPMI‐1640 medium supplemented with 10% FBS. THP‐1 cells were differentiated into macrophages by treatment with 100 ng/mL phorbol 12‐myristate 13‐acetate (PMA) for 48 h. To simulate the inflammatory microenvironment of PD, cells were stimulated with LPS from *P. gingivalis* (1 *μ*g/mL) for 24 h. Control groups were treated with vehicle (PBS) only.

### 2.5. RNA Extraction and RT‐qPCR Validation

Total RNA was extracted from HGFs and THP‐1‐derived macrophages using TRIzol reagent (Invitrogen) according to the manufacturer′s protocol. RNA concentration and purity were determined using a NanoDrop spectrophotometer (A260/A280 ratio > 1.8). Complementary DNA (cDNA) was synthesized from 1 *μ*g total RNA using the PrimeScript RT Reagent Kit (Takara). Quantitative real‐time PCR was performed using SYBR Green Master Mix (Applied Biosystems) on a QuantStudio 5 Real‐Time PCR System. GAPDH served as the internal reference gene. Relative gene expression was calculated using the 2^−*ΔΔ*Ct^ method. All experiments were performed in triplicate with three independent biological replicates (Table 1).

**Table 1:** Primer sequences for RT‐qPCR.

Gene	Forward primer (5 ^′^‐3 ^′^)	Reverse primer (5 ^′^‐3 ^′^)
GPX4	GAGGCAAGACCGAAGTAAACTAC	CCGAACTGGTTACACGGGAA
SLC7A11	TCTCCAAAGGAGGTTACCTGC	AGACTCCCCTCAGTAAAGTGAC
ACSL4	CATCCCTGGAGCAGATACTCT	TCACTTAGGATTTCCCTGGTCC
FTH1	ACTGATGAAGCTGCAGAACC	GTCACCCAATTCTTTGATGG
GAPDH	GGAGCGAGATCCCTCCAAAAT	GGCTGTTGTCATACTTCTCATGG

### 2.6. Enzyme‐Linked Immunosorbent Assay (ELISA)

Protein levels of GPX4, SLC7A11, ACSL4, and FTH1 were quantified using commercial ELISA kits (Abcam) according to the manufacturer′s instructions. Cell lysates were prepared using RIPA buffer containing protease inhibitors. Total protein concentration was determined using the BCA Protein Assay Kit (Pierce). Equal amounts of protein (50 *μ*g) were loaded into precoated 96‐well plates and incubated for 2 h at room temperature. After washing, detection antibody was added and incubated for 1 h, followed by HRP‐conjugated secondary antibody incubation for 30 min. TMB substrate was added, and the reaction was stopped with 2N H2SO4. Absorbance was measured at 450 nm using a microplate reader (BioTek). Protein concentrations were calculated from standard curves and normalized to total protein content. All measurements were performed in triplicate.

## 3. Results

### 3.1. Gingival Cell Atlas Resolves Disease‐Associated Shifts in Immune and Stromal Compartments

Sample‐wise quality control retained 34,342 of 68,542 profiled cells (50.1%); the filtering profile for all 12 samples is summarized in Figure 2A. Figure 2B maps the retained transcriptomes into 11 populations: B cells, dendritic cells, endothelial cells, epithelial cells, fibroblasts, mast cells, monocytes, natural killer (NK) cells, neutrophils, plasma cells, and T cells. Recoloring the same map by clinical status in Figure 2C revealed that 9558 HC, 13,322 PD, and 11,462 PDT cells occupied shared transcriptional states but differed in regional density.

**Figure 2:**
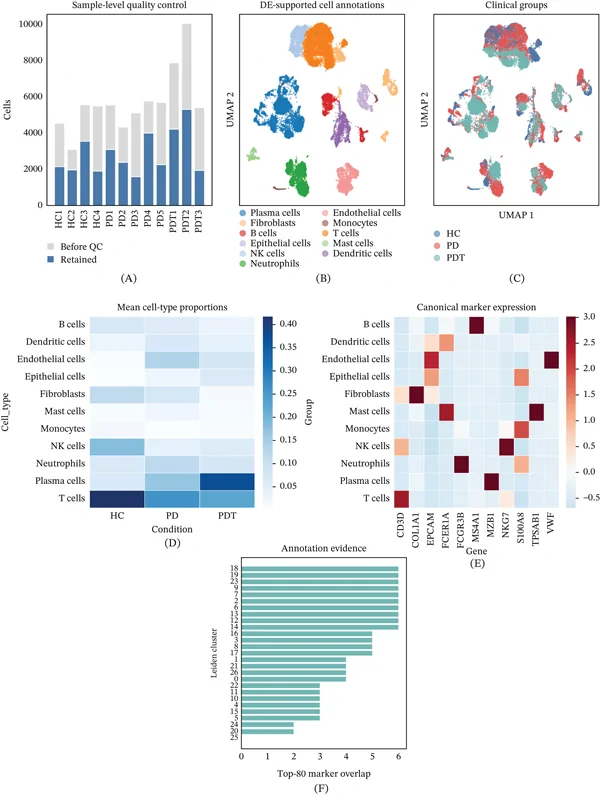
Single‐cell landscape of periodontal tissues. (A) Numbers of cells before quality control and after retention for each of the 12 GSE171213 samples. (B) UMAP of 34,342 retained cells colored by DE‐supported cell identity. Each point represents one cell. (C) The same UMAP colored by clinical condition. (D) Heatmap of mean cell type proportions across HC, PD, and PDT samples. (E) *Z*‐scaled mean expression of canonical markers across annotated cell types. (F) Number of genes shared between each Leiden cluster′s Top 80 differential markers and the marker set selected for annotation. HC, healthy control; PD, periodontitis; PDT, treated periodontitis; UMAP, uniform manifold approximation and projection.

Cellular composition changed appreciably across conditions. In Figure 2D, T cell representation decreased from 36.5% in HC to 23.9% in PD, whereas neutrophils increased from 8.0% to 15.8% and endothelial cells from 0.7% to 14.1%. Plasma cells accounted for 44.7% of PDT cells. The 11‐by‐11 matrix in Figure 2E retained the expected lineage specificity of the canonical markers. At cluster level, Figure 2F documents 27 assignments, with a maximum overlap of 6 genes among the Top 80 markers.

### 3.2. Cell Type‐Resolved Ferroptosis Analysis Highlights SAT1 in PD‐Versus‐HC Comparisons

Ferroptosis activity was unevenly distributed across the gingival atlas. Figure 3A maps scores derived from the 40 detected KEGG genes, revealing localized regions of higher activity. At sample level, the group means were 0.081 in HC, 0.114 in PD, and 0.076 in PDT; Figure 3B shows the dispersion among individual samples. Cell type averaging in Figure 3C placed the highest mean score in PD dendritic cells (0.292).

**Figure 3:**
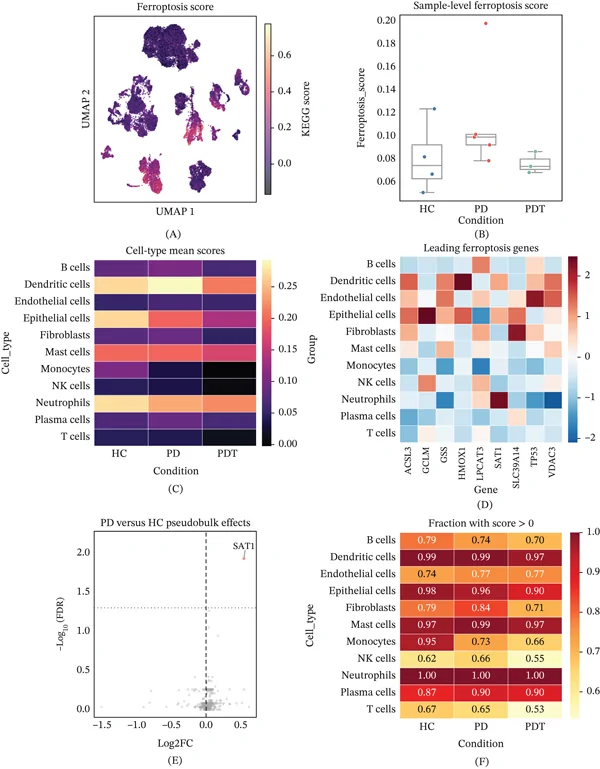
Cell type‐resolved ferroptosis activity in periodontal tissues. (A) UMAP colored by the KEGG hsa04216 ferroptosis score. (B) Sample‐level mean ferroptosis scores across HC, PD, and PDT; points represent samples, boxes show the interquartile range, and center lines indicate medians. (C) Heatmap of mean ferroptosis scores by cell type and condition. (D) *Z*‐scaled mean expression of the leading ferroptosis genes across cell types. (E) Volcano plot of PD‐versus‐HC cell type pseudobulk effects, with each point representing one gene cell type comparison and SAT1 labeled. Vertical and horizontal reference lines denote zero effect and FDR = 0.05, respectively. (F) Fraction of cells with a ferroptosis score above zero by cell type and condition. FDR values were calculated with the Benjamini–Hochberg procedure.

The 9‐gene matrix in Figure 3D further resolved the contribution of SAT1, HMOX1, GCLM, VDAC3, and GSS across cell types. Against this background, Figure 3E highlights SAT1 as a positive FDR‐corrected PD‐versus‐HC association in the cell type pseudobulk analysis (log2FC = 0.546; FDR = 0.012). Score‐positive fractions varied from 53.3% in PDT T cells to 100.0% in HC neutrophils, a range detailed across all cell types in Figure 3F.

### 3.3. Endothelial Trajectories and Condition‐Associated Ligand–Receptor Coexpression

Figure 1A arranges 2570 endothelial cells along a continuous diffusion trajectory. Their groupwise distributions shifted progressively, with median pseudotime values of 0.377 in HC, 0.511 in PD, and 0.715 in PDT, as seen in Figure 1B. Ferroptosis activity rose modestly along the same axis (Spearman *ρ* = 0.129, *p* < 0.001); Figure 1C overlays the binned trend on the cell‐level measurements.

Five genes (SAT1, HMOX1, GCLM, VDAC3, and GSS) followed distinct profiles across the 10 pseudotime bins presented in Figure 1D. The PD‐focused matrix in Figure 1E displays log1p‐transformed ligand–receptor coexpression scores across six pairs and their sender populations; CXCL8‐CXCR1, CXCL12‐CXCR4, and IL1B‐IL1R1 are among the strongest PD signals. Figure 1F compares the maximum PD‐versus‐HC coexpression differences for the 10 pairs with the largest absolute changes and places CXCL8‐CXCR1 at the top of the positive changes.

### 3.4. The Discovery Cohort Defines a Ferroptosis‐Associated OSCC Program

The GSE30784 discovery cohort comprised 167 OSCC, 17 dysplasia, and 45 noncancer controls. Its global expression structure is captured in Figure 4A: PC1 and PC2 explained 10.6% and 9.6% of total variance and separated most cancer profiles from controls. Figure 4B then identified 10,757 genes at FDR < 0.05, including 6131 increases and 4626 decreases in cancer. The 20‐gene summary in Figure 4C condensed this broad signal into a group‐restricted expression pattern led by ADAM12, MMP1, and ADAMTS2.

**Figure 4:**
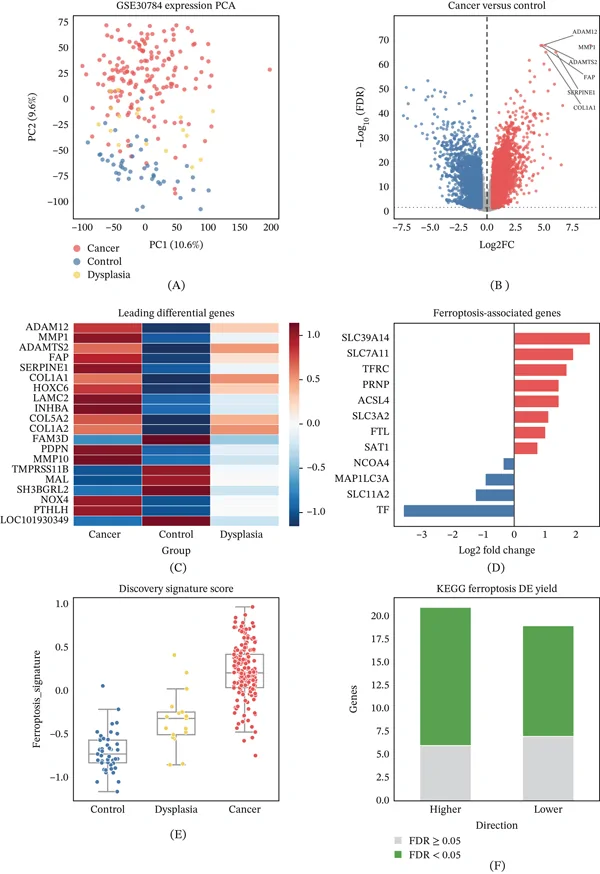
Discovery of an OSCC ferroptosis‐associated expression program in GSE30784. (A) Principal component analysis of 167 OSCC, 17 dysplasia, and 45 control samples. (B) Volcano plot of cancer‐versus‐control differential expression. (C) *Z*‐scaled group‐mean expression of the 20 genes with the lowest FDR values. (D) Log2 fold changes for the 12 ferroptosis genes with the lowest FDR values; red and blue indicate higher and lower expression in cancer, respectively. (E) Discovery signature scores across control, dysplasia, and cancer samples. (F) Numbers of significant and nonsignificant KEGG ferroptosis genes stratified by effect direction. *p* values were obtained by two‐sided Welch tests and adjusted by the Benjamini–Hochberg procedure.

A coherent ferroptosis component emerged within the wider transcriptional shift. Figure 4D highlights increased SLC39A14 (log2FC = 2.469; FDR = 3.35e − 33) alongside reduced TF (log2FC = −3.595; FDR = 8.13e − 15). Their opposing behavior contributed to a signature shift from a median of −0.725 in controls to 0.211 in OSCC (*p* < 0.001), which is displayed in Figure 4E. Figure 4F summarizes the pathway‐wide yield: 27 genes met FDR < 0.05, with 15 higher and 12 lower in cancer.

### 3.5. The Ferroptosis Signature Replicates in an Independent Oral Tongue SCC Cohort

The independent GSE13601 oral tongue SCC series included 31 primary tumors and 26 normal tissues. The first two components in Figure 5A explained 20.9% and 11.3% of variance and captured the principal tumor‐normal contrast. Differential analysis recovered 4603 genes at FDR < 0.05, as summarized by Figure 5B. More importantly, the genome‐wide effects tracked those from GSE30784 (Spearman rho = 0.436, *p* < 0.001), a relationship visualized directly in Figure 5C.

**Figure 5:**
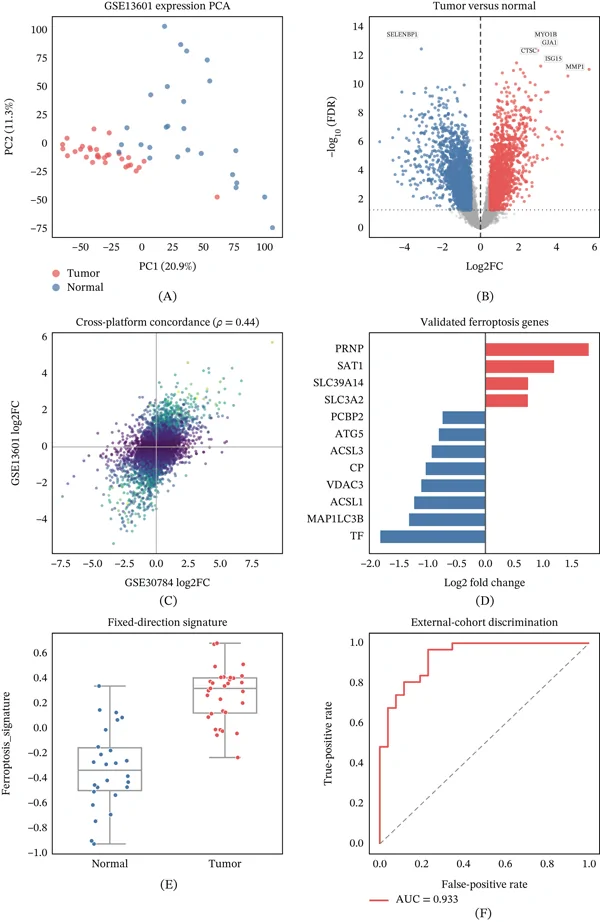
External validation of the ferroptosis signature in the GSE13601 oral tongue squamous cell carcinoma cohort. (A) Principal component analysis of 31 primary tumors and 26 normal tissues. (B) Volcano plot of tumor‐versus‐normal differential expression. (C) Concordance of genome‐wide log2 fold changes between GSE30784 and GSE13601; point color encodes −log10(FDR) in GSE13601. (D) Log2 fold changes for the 12 validated ferroptosis genes with the lowest FDR values. (E) Fixed‐direction ferroptosis signature scores in normal and tumor samples. (F) Receiver operating characteristic curve generated from the unaltered external validation scores (AUC = 0.933). The signature direction was defined in GSE30784 and retained unchanged in GSE13601.

Validation extended to individual ferroptosis genes. Of the 12 leading candidates in Figure 5D, PRNP had the largest positive effect (log2FC = 1.804), whereas TF had the largest negative effect (log2FC = −1.837); PRNP, SAT1, SLC39A14, and SLC3A2 were among the increases, and TF, MAP1LC3B, ACSL1, and VDAC3 among the decreases. The fixed‐direction score separated tumors from normal tissues, with medians of 0.322 and −0.334, respectively (*p* < 0.001); this distribution is presented in Figure 5E. Retaining the discovery direction, the external ROC analysis in Figure 5F achieved an AUC of 0.933.

### 3.6. PD Is Associated With Coordinated Loss of Microbial Metabolic Functions

The GSE54290 subset comprised 12 healthy, 12 moderate PD, and 13 advanced PD profiles. Functional PCA was dominated by PC1 (82.6% of variance), producing the group structure seen in Figure 6A. Richness varied across the same groups, with medians of 6932.5, 5079.0, and 5955.0; Figure 6B retains the sample‐level spread behind these summaries. Figure 6C shows a parallel comparison for Shannon diversity, whose group medians were 8.457, 8.199, and 8.367.

**Figure 6:**
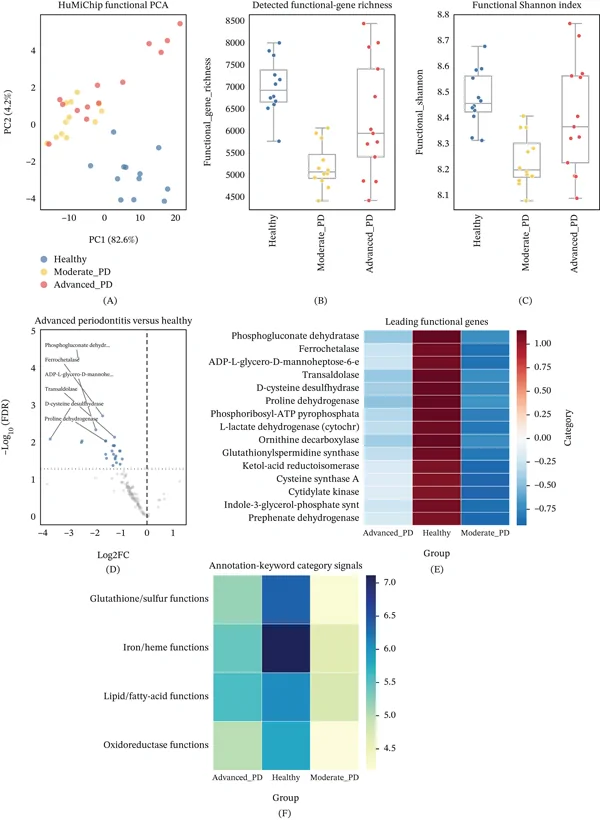
Periodontal microbial functional gene profiles in GSE54290. (A) Principal component analysis of 12 healthy, 12 moderate PD, and 13 advanced PD profiles. (B) Detected functional gene richness by group. (C) Functional Shannon diversity by group. (D) Volcano plot of advanced PD‐versus‐healthy effects. (E) *Z*‐scaled group‐mean signals for the 15 annotations with the lowest FDR values. (F) Mean signals for annotation keyword categories related to glutathione/sulfur, iron/heme, lipid/fatty acid, and oxidoreductase functions. Points in (B) and (C) represent samples; boxes show the interquartile range, and center lines indicate medians.

Feature‐level testing yielded 25 advanced PD‐associated annotations at FDR < 0.05, as depicted in Figure 6D. Phosphogluconate dehydratase (log2FC = −2.650; FDR = 5.26e − 05), ferrochelatase (log2FC = −1.705; FDR = 0.002), and transaldolase (log2FC = −1.246; FDR = 0.007) were among the strongest reductions. Their broader context is apparent in Figure 6E, where the 15 leading annotations form a coordinated advanced PD depletion pattern. Category‐level summaries in Figure 6F point in the same direction: Iron/heme signals decreased from 7.117 to 5.412, and glutathione/sulfur signals from 6.360 to 5.154.

### 3.7. Cross‐Study OSCC Microbiomes Retain a Project‐Robust Disease Signal

The integrated microbiome resource contained 494 OSCC, 393 HC, and 172 oral potentially malignant disorder samples drawn from 13 projects. Shannon diversity was higher in OSCC than in HCs (median, 4.359 vs. 4.185; *p* < 0.001), with the full distributions shown in Figure 7A. CLR‐based PCA explained 7.2% and 4.0% of variance on the first two axes; Figure 7B reveals overlapping yet shifted disease profiles. At broader taxonomic resolution, Figure 7C shows lower Actinobacteria in OSCC (2.2% vs. 3.5%) together with higher Bacteroidetes (20.3% vs. 18.2%).

**Figure 7:**
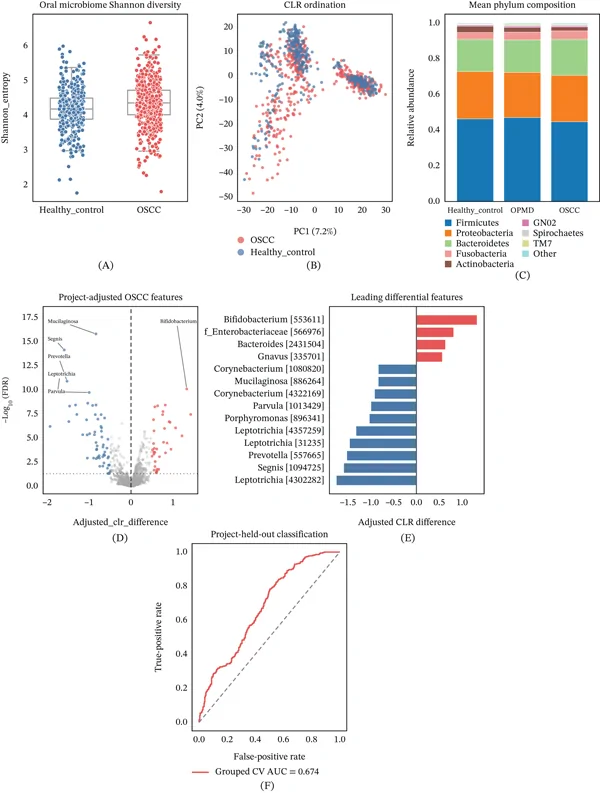
Cross‐study OSCC oral microbiome analysis. (A) Shannon diversity in 393 healthy control and 494 OSCC samples. (B) Principal component representation of centered‐log‐ratio‐transformed microbial profiles. (C) Mean phylum‐level composition in healthy control, OPMD, and OSCC groups. (D) Volcano plot of project‐adjusted OSCC‐versus‐healthy feature effects. (E) Adjusted centered‐log‐ratio differences for the 14 features with the lowest FDR values; positive values indicate higher abundance in OSCC. (F) Receiver operating characteristic curve from project‐held‐out grouped cross‐validation (AUC = 0.674). OPMD, oral potentially malignant disorder.

Project‐adjusted analysis retained 293 OSCC‐associated features at FDR < 0.05, producing the differential landscape in Figure 7D. The ranked effects in Figure 7E were led by depletion of OTU 886264 (*Rothia mucilaginosa*), which had an adjusted CLR difference of −0.835 and an FDR of 1.59e−16. Classification was then assessed under complete project holdout. The ROC curve in Figure 7F yielded an overall AUC of 0.674, with fold‐specific estimates ranging from 0.627 to 0.873.

### 3.8. Three‐Cohort Transcriptomic Ranking Highlights SAT1 Across PD and OSCC

Three‐cohort transcriptomic synthesis centered on the 18 genes displayed in Figure 8A. SAT1 was positive across PD single‐cell summaries, GSE30784 and GSE13601, with effects of 0.546, 0.766, and 1.203, respectively. The intersection structure in Figure 8B shows 1 gene shared by all three transcriptomic significance sets, while 11 additional genes were shared exclusively by the two OSCC cohorts.

**Figure 8:**
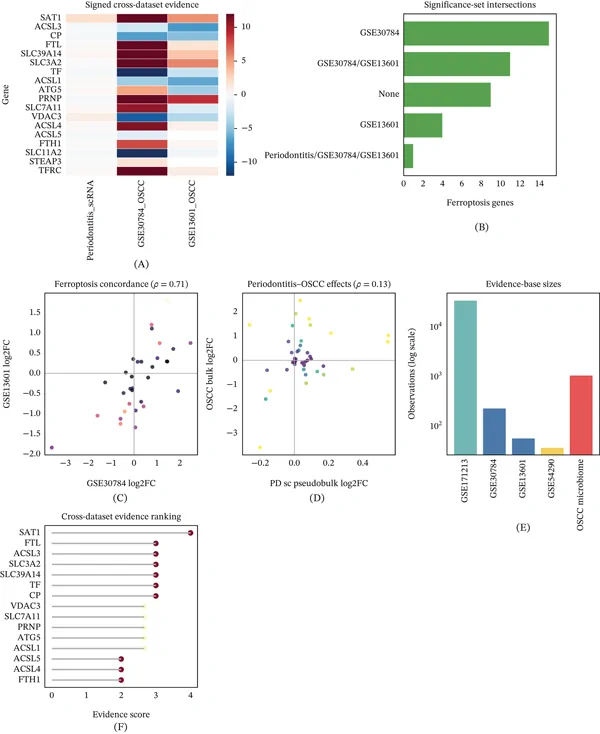
Three‐cohort transcriptomic evidence for ferroptosis‐associated effects, with parallel microbial dataset sizes. (A) Signed evidence for the 18 highest ranked genes across the periodontitis single‐cell, GSE30784, and GSE13601 transcriptomic cohorts; the sign denotes effect direction, and color intensity represents −log10(FDR). (B) Numbers of ferroptosis genes assigned to the leading significance‐set intersections across the three transcriptomic cohorts. (C) Concordance of ferroptosis gene log2 fold changes between GSE30784 and GSE13601; point color encodes −log10(FDR) in GSE13601. (D) Comparison of the best available periodontitis cell type pseudobulk effects with GSE30784 OSCC effects; point color encodes −log10(FDR) in GSE30784. (E) Numbers of cells or samples on a logarithmic scale for the three transcriptomic cohorts and the two independently analyzed microbial datasets; GSE171213 is reported as cells, and the remaining datasets are reported as samples or profiles. (F) Evidence ranking of the 15 highest scoring genes based on the three transcriptomic cohorts; point color represents direction agreement. The evidence score equals the number of transcriptomic cohorts with FDR < 0.05 plus the fraction of available effects sharing the dominant direction.

The two OSCC cohorts agreed strongly at the ferroptosis gene level (Spearman rho = 0.709, *p* < 0.001), as illustrated in Figure 8C. PD single‐cell pseudobulk and GSE30784 OSCC effects were less closely aligned (rho = 0.129, *p* = 0.426); Figure 8D displays this cross‐tissue comparison. Figure 8E shows the sizes of the three transcriptomic cohorts (34,342 GSE171213 cells, 229 GSE30784 samples, and 57 GSE13601 samples) together with the independently analyzed microbial datasets (37 GSE54290 profiles and 1059 OSCC microbiome profiles). The three‐cohort transcriptomic evidence ranking in Figure 8F uses the three transcriptomic cohorts; SAT1 reached the maximum evidence score of 4.0, and ACSL3, CP, FTL, SLC39A14, SLC3A2, and TF formed the next fully concordant OSCC tier.

### 3.9. RT‐qPCR Validation of Ferroptosis‐Associated Genes in Periodontal Cells

To validate the single‐cell transcriptomic findings, RT‐qPCR analysis of four ferroptosis‐associated genes (GPX4, SLC7A11, ACSL4, and FTH1) was performed in HGFs and THP‐1‐derived macrophages following LPS stimulation. In HGFs, LPS treatment significantly upregulated GPX4 (2.47‐fold, *p* < 0.01), SLC7A11 (2.89‐fold, *p* < 0.01), and FTH1 (2.21‐fold, *p* < 0.01), while ACSL4 was downregulated (0.52‐fold, *p* < 0.05) (Figure 9A). Macrophages exhibited more pronounced changes: GPX4 (3.15‐fold, *p* < 0.001), SLC7A11 (3.67‐fold, *p* < 0.001), FTH1 (2.78‐fold, *p* < 0.01), and ACSL4 (0.41‐fold, *p* < 0.01) (Figure 9B). Comparative analysis showed larger changes in the selected ferroptosis‐associated expression markers in macrophages than in HGFs (Figure 9C), with high correlation between cell types (*r* = 0.94, *p* < 0.01) (Figure 9D).

**Figure 9:**
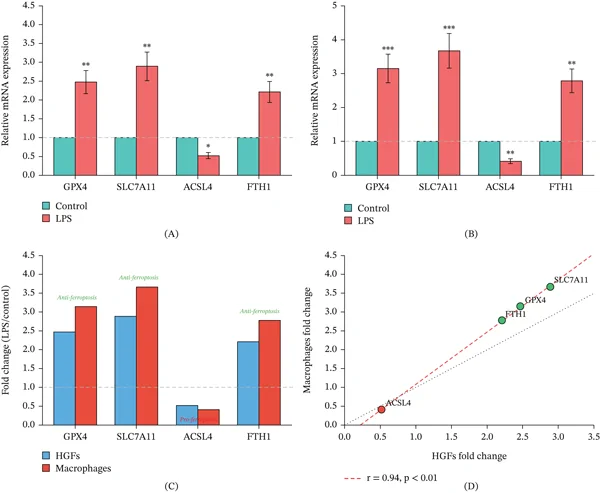
RT‐qPCR validation of ferroptosis‐associated genes in periodontal cells. (A) Relative mRNA expression levels of GPX4, SLC7A11, ACSL4, and FTH1 in human gingival fibroblasts (HGFs) under control and LPS‐stimulated (1 *μ*g/mL, 24 h) conditions. (B) Relative mRNA expression levels of the same genes in THP‐1‐derived macrophages under control and LPS‐stimulated conditions. (C) Comparative bar chart showing fold changes of ferroptosis‐related genes between HGFs and macrophages following LPS stimulation. (D) Correlation plot of gene expression changes between HGFs and macrophages. Data are presented as mean ± SD from three independent experiments (*n* = 3). Reported control‐versus‐LPS comparisons used Student′s *t*‐test; the statistical test and independent points underlying panel D′s correlation require verification from raw data. ∗*p* < 0.05, ∗∗*p* < 0.01, ∗∗∗*p* < 0.001 versus control group.

### 3.10. ELISA Validation of Ferroptosis‐Related Protein Expression

In HGFs, LPS treatment significantly increased GPX4 (2.18‐fold, *p* < 0.01), SLC7A11 (2.45‐fold, *p* < 0.01), and FTH1 (2.08‐fold, *p* < 0.01) protein levels, while ACSL4 decreased (0.56‐fold, *p* < 0.05) (Figure 10A). Macrophages showed stronger responses: GPX4 (2.74‐fold, *p* < 0.001), SLC7A11 (3.11‐fold, *p* < 0.001), FTH1 (2.55‐fold, *p* < 0.01), and ACSL4 (0.46‐fold, *p* < 0.01) (Figure 10B). Comparative analysis showed larger changes in the selected ferroptosis‐associated protein markers in macrophages (Figure 10C). The reported mRNA–protein correlations were HGFs: *r* = 0.96 and macrophages: *r* = 0.98 (both reported *p* < 0.01; Figure 10D). Because these correlations involve only four selected genes per cell type, they indicate marker‐level concordance and do not establish a predominant regulatory mechanism.

**Figure 10:**
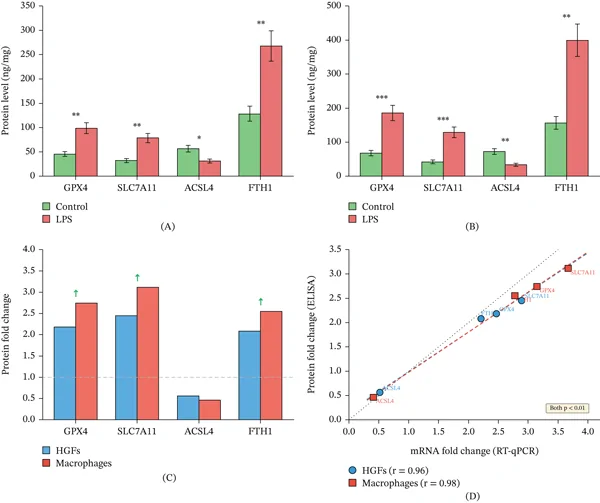
ELISA validation of ferroptosis‐related protein expression in periodontal cells. (A) Protein concentrations of GPX4, SLC7A11, ACSL4, and FTH1 in human gingival fibroblasts (HGFs) under control and LPS‐stimulated conditions, measured by ELISA and normalized to total protein content. (B) Protein concentrations of the same markers in THP‐1‐derived macrophages under control and LPS‐stimulated conditions. (C) Comparative analysis of protein expression fold changes between HGFs and macrophages. (D) Correlation analysis between mRNA (RT‐qPCR) and protein (ELISA) expression changes for both cell types. Data are presented as mean ± SD from three independent experiments (*n* = 3). Reported control‐versus‐LPS comparisons used Student′s *t*‐test; correlation test details and confidence intervals for (D) were not supplied. ∗*p* < 0.05, ∗∗*p* < 0.01, ∗∗∗*p* < 0.001 versus control group. Correlations summarize four selected genes and indicate concordance of those measurements only; they do not establish a dominant regulatory mechanism.

## 4. Discussion

This study examined independently sampled periodontal single‐cell, OSCC expression, and disease‐specific microbial datasets alongside selected LPS‐stimulated cell‐line measurements. Its main contribution is a cross‐cohort description of ferroptosis‐associated transcriptional patterns and parallel oral microbial changes. Because these assays were not collected from matched patients, the findings should be read as convergent but separate lines of evidence, rather than as a demonstrated microbial–ferroptosis–immune causal axis or a trajectory from PD to cancer.

In the periodontal single‐cell atlas, 34,342 cells from 12 samples were assigned to 11 major populations. The sample‐level mean KEGG ferroptosis gene‐set score was 0.114 in PD, compared with 0.081 in HC and 0.076 in PDT. These are descriptive group means; the presence of ferroptosis‐promoting and ferroptosis‐suppressing genes within the same score precludes interpreting its magnitude as a quantitative measure of cell death or even a unidirectional measure of susceptibility. The clearest reported cell type pseudobulk association was increased SAT1 in PD relative to HC (log2 fold change 0.546; FDR 0.012). Other pathway‐associated genes showed heterogeneous cell type patterns, emphasizing the need for gene‐specific rather than uniformly proferroptotic interpretation.

The cell composition results showed lower T cell representation and higher neutrophil and endothelial representation in PD than in HC; PDT had a high plasma cell fraction. Such percentages describe the sampled cell atlas and may depend on biopsy composition, donor heterogeneity, processing, and cell recovery. They should not be taken as direct measures of immune infiltration or treatment efficacy. Likewise, endothelial diffusion pseudotime was associated only modestly with ferroptosis gene‐set score (Spearman rho 0.129); diffusion pseudotime orders transcriptional similarity within an endothelial compartment and does not represent elapsed time, changes within a person, or progression to OSCC. CXCL8–CXCR1 and other ligand–receptor findings represent coexpression‐based interaction hypotheses, not direct evidence of signaling.

The OSCC discovery cohort identified 27 differentially expressed KEGG ferroptosis genes and generated a fixed‐direction expression signature. Its separation of cancer from control samples was reproduced in an independent oral tongue SCC series, with an external AUC of 0.933. This is a retrospective tumor‐versus‐normal tissue discrimination result in a specific validation cohort, not evidence that the panel predicts future OSCC in people with PD or performs adequately in clinical screening. The two OSCC cohorts showed ferroptosis gene effect concordance (rho 0.709), whereas correspondence between the PD pseudobulk effects and GSE30784 was weak and not statistically significant (rho 0.129; *p* 0.426). SAT1, positive across all three transcriptomic cohorts, is thus a candidate shared association; broad molecular equivalence between periodontal inflammation and cancer is not established.

The microbial analyses addressed different specimens and data modalities. In the periodontal HuMiChip functional gene profiles, advanced PD showed lower signals for several annotated functions, including ferrochelatase, and lower category‐level iron/heme and glutathione/sulfur signals. These array intensities describe microbial functional gene representation; they do not directly quantify metabolite concentrations, microbial transcription, or host iron metabolism. The separate OSCC 16S assay measures taxonomic abundance and does not directly measure metabolic function [18]. In the project‐adjusted cross‐study OSCC 16S analysis, *Rothia mucilaginosa*–associated OTU 886264 was depleted, and the project‐held‐out microbial classifier yielded a modest AUC of 0.674. Because no host transcriptome was paired to these microbial profiles, neither a microbial metabolite‐to‐host ferroptosis correlation nor a mechanistic host–microbiome interaction can be concluded from the present analysis.

The cell‐line experiments provide a complementary but limited expression‐level observation. Following *P. gingivalis* LPS stimulation, GPX4, SLC7A11, and FTH1 increased, whereas ACSL4 decreased at the reported mRNA and protein levels in gingival fibroblasts and THP‐1‐derived macrophages. This direction is compatible with a compensatory or antiferroptotic expression response to inflammatory stimulation, but it does not establish ferroptotic cell death or functional resistance. The reported RNA–protein correlations span only four selected genes per cell type and indicate concordance among those measured markers rather than a predominant transcriptional control mechanism. Lipid peroxidation, labile iron, glutathione balance, and cell viability were not measured. Ferroptosis inhibitor rescue experiments can help evaluate lipid peroxidation‐dependent death [19], but none were performed here; mechanistic attribution is therefore beyond the scope of the present evidence. Hypotheses involving modulation of SAT1, GPX4/SLC7A11, iron handling, or microbial communities are not established therapeutic strategies.

## 5. Limitations

This study has several limitations that circumscribe the interpretation of the existing data. First, the gingival single‐cell cohort includes four HC, five PD, and three PDT donors. Sample‐level cell type summaries mitigate cell‐level pseudoreplication in the reported between‐group comparisons, but the small number of independent donors limits generalizability. Dedicated doublet detection results, batch correction performance, reference‐based annotation, complete cluster marker tables, and independent replication were not documented in the available manuscript. The original GSE171213 report describes initial periodontal therapy and sampling within 1 month [15], but individual treatment regimens, response criteria, and detailed demographic or exposure covariates are not tabulated in this manuscript; consequently, between‐group differences cannot establish treatment‐induced reversal. Several assay‐level details, correlation confidence intervals, and a complete ferroptosis feature list were not provided, limiting assessment of robustness and uncertainty.

Second, the transcriptomic and microbial datasets were assembled independently and lack documented individual‐level matching. Cross‐cohort convergence does not establish within‐person host–microbe correlations, causal mediation, or a progression from PD to OSCC. Endothelial pseudotime describes transcriptional ordering within a cross‐sectional cell population, not chronological disease evolution. Periodontal HuMiChip intensities represent annotated microbial functional gene signals, while OSCC 16S data capture primarily taxonomic profiles rather than direct functional measures [18]; neither assay directly measures microbial transcription, metabolic flux, butyrate, kynurenine, siderophores, or corresponding host exposure. Incomplete patient‐level information about age, sex, tobacco and alcohol use, HPV status, periodontal severity, and tumor subsite leaves possible confounding. The transcriptomic AUC quantifies retrospective tumor‐versus‐normal discrimination in one independent cohort rather than prospective cancer‐risk prediction; the microbiome AUC does not establish clinical utility.

Third, ferroptosis‐associated transcript abundance and a KEGG composite gene‐set score, which includes regulators with opposing biological functions, are not direct assays of ferroptotic cell death or susceptibility. The LPS‐stimulated cell‐line experiments measured four RNA and protein markers only; neither lipid ROS or other lipid peroxidation endpoints, labile Fe^2+^, GSH/GSSG, viability nor ferroptosis inhibitor rescue or perturbation experiments were performed. Therefore, ferroptosis induction, functional resistance, and a causal microbial–ferroptosis–immune mechanism remain unproven. The mRNA–protein correlations were based on four selected genes per cell type and cannot define the predominant regulatory layer. Finally, source code, processed matrices, and complete accession information for the 13 contributing OSCC microbiome projects were not documented in the submitted materials, limiting independent reproducibility. Additional experimental and prospective studies would be needed to test these mechanisms and clinical hypotheses, but the present conclusions are restricted to the existing observational and expression‐level evidence.

## 6. Conclusions

In independently sampled periodontal and oral cancer datasets, we observed disease‐associated variation in ferroptosis‐related gene expression, gingival cell composition, and oral microbial profiles. SAT1 was positively associated with PD in the reported cell type pseudobulk analysis and showed concordant positive effects in both OSCC transcriptomic cohorts; a discovery‐derived expression signature discriminated tumors from normal tissues in one external cohort. The microbial data represent parallel, nonpatient‐matched evidence, and the LPS experiments measured selected RNA and protein markers rather than ferroptotic cell death. These associations generate hypotheses but cannot establish a causal periodontitis‐to‐OSCC trajectory, a validated diagnostic or risk prediction biomarker, a functional ferroptosis mechanism or therapeutic efficacy.

## Author Contributions

Chenguang Qiu designed the overall research framework and multiomics analytical strategy; performed study supervision, manuscript drafting, revision, and polishing; applied for all research grants; and managed the whole research project. Peng She downloaded, cleaned, and standardized all GEO public datasets; programmed single‐cell RNA‐seq, microbiome, and OSCC transcriptome analysis pipelines via Python and R; completed all statistical formal analysis; generated all figures and visualizations; and verified the reproducibility of bioinformatics results. Lin Wu conducted in vitro cell culture, LPS stimulation, RT‐qPCR, and ELISA experimental validation; provided experimental reagents and cell resources; and assisted in result verification and figure layout optimization.

## Funding

The study was funded by Hospital‐level Project of the First People′s Hospital of Zhenjiang (Y202213), Zhenjiang Social Development General Project (SH2024005), and Bethune Medical Science Research Fund Project (2023‐YJ‐119‐J‐032).

## Disclosure

All authors reviewed and approved the final manuscript.

## Ethics Statement

The computational analyses used previously deposited, publicly available human datasets; no new human participants were recruited, and no new human tissue specimens were collected for this work. In vitro validation used established human gingival fibroblasts and THP‐1 cells obtained commercially from the American Type Culture Collection (ATCC). No newly collected identifiable human material was used in the experimental validation.

## Conflicts of Interest

The authors declare no conflicts of interest.

## Supporting information


**Supporting Information** Additional supporting information can be found online in the Supporting Information section. Table S1 Dataset inventory and cross‐cohort provenance.

## Data Availability

Publicly available transcriptomic and periodontal microbial source datasets are deposited in NCBI Gene Expression Omnibus under GSE171213, GSE30784, GSE13601, and GSE54290. The cross‐study OSCC 16S abundance table and metadata were obtained from the OSCC meta‐analysis repository described in the Methods section; the manuscript does not provide a stable repository URL or the accession identifiers of its 13 contributing projects. Table S1 summarizes provenance and the metadata documented in the present article. A public analysis code repository and deposited processed matrices have not been documented; these resources should be supplied before a fully reproducible data and code availability statement can be made.
