# Supplementary material for: Cross‐Cohort Transcriptomic and Oral Microbiome Analyses Identify Ferroptosis‐Associated Signatures in Periodontitis and Oral Squamous Cell Carcinoma
Source: Int J Genomics. 2026 Nov 3;2026:7538825. doi: 10.1155/ijog/7538825 (PMC13732103; doi:10.1155/ijog/7538825)
Supplement: Supplementary file 1 [file IJOG-2026-7538825-s001.docx]

# Supplementary Table S1. Dataset inventory and cross-cohort provenance

Information below is based on the submitted manuscript and, where explicitly cited, the original GSE171213 publication. “Not reported” indicates information that must be retrieved from the original dataset record or authors; it is not assumed to be absent from the underlying repository. The datasets are analyzed in parallel and no individual-level cross-modal matching is documented.

| **Dataset / resource** | **N and groups** | **Specimen / assay / platform** | **Covariates reported here** | **Analytic role and linkage** |
| --- | --- | --- | --- | --- |
| GSE171213 | 12 donors: 4 HC, 5 PD, 3 PDT; 34,342 retained cells | Periodontal tissue; scRNA-seq; BD Rhapsody; GEO platform GPL24676 [15] | HC, severe chronic PD and severe chronic PD following initial periodontal therapy (within 1 month) described in source study [15]; detailed per-person exposures and response criteria not tabulated here | Cell atlas; cell-type pseudobulk; score and trajectory analyses. No patient matching to other resources documented. |
| GSE30784 | 229: 167 OSCC, 17 dysplasia, 45 non-cancer controls | Oral tissue expression microarray; GPL570 | Diagnostic groups; age, sex, smoking, subsite, HPV and other covariates not tabulated | OSCC discovery differential expression and ferroptosis signature; independent of periodontal dataset. |
| GSE13601 | 57: 31 oral tongue SCC, 26 normal tissues | Oral tongue tissue expression microarray; GPL8300 | Diagnostic groups and oral tongue site; patient-level covariates not tabulated | Independent external expression-signature validation; no individual matching documented. |
| GSE54290 | 37: 12 healthy, 12 moderate PD, 13 advanced PD | Periodontal microbial functional-gene profiles; HuMiChip; GPL18196 | Disease severity groups; age, sex, smoking and treatment metadata not tabulated | Independent periodontal microbial functional-gene comparisons; no host expression matching documented. |
| OSCC-meta-analysis repository | 1,059 profiles: 494 OSCC, 393 healthy, 172 potentially malignant disorder; 13 projects | Oral microbiome 16S abundance table; ConQuR-adjusted; Greengenes 13.8 taxonomy; original platforms not individually reported | Diagnosis and project used in adjustment; subject-level clinical covariates and contributing accessions not listed | Separate OSCC taxonomic analysis and group-project-held-out classifier; no patient matching to host expression documented. |

# Supplementary Note S1. Analytical hierarchy and workflow

Reporting hierarchy (assigned for this revision; no prospective preregistration is claimed): primary—periodontal cell identities and ferroptosis-associated transcriptional differences; secondary—OSCC discovery/independent validation and separate microbial profiles; exploratory—endothelial pseudotime, ligand–receptor coexpression, and cross-cohort candidate ranking.

Workflow: GSE171213 → single-cell QC → annotation → per-sample cell-type summaries and KEGG score → PD-versus-HC association; GSE30784 → OSCC signature discovery → fixed-direction testing in GSE13601; GSE54290 → periodontal functional-gene comparisons; cross-study OSCC 16S → project-adjusted taxonomic associations and project-held-out classification; independent outputs → descriptive cross-cohort synthesis. Cell-line RT-qPCR and ELISA separately assess selected expression responses.

Inferential boundaries: Sample-level expression associations are not cell-death assays; single-cohort pseudotime is not longitudinal progression; independent host and microbial cohorts cannot support patient-level mediation; retrospective tissue AUC is not prospective clinical risk prediction.

Information not documented in the supplied materials includes the complete KEGG feature list, donor-level clinical covariates, per-cluster marker tables, validation raw points and confidence intervals, accession identifiers for all contributing 16S projects, scripts/notebooks and processed analysis objects. These omissions are disclosed as limitations of reproducibility and inference; no additional experiment, supplementary analysis or public deposit is claimed.
